# A Qualitative Study Into the Relative Stigmatization of Mental Illness by Mental Health Professionals

**DOI:** 10.1177/10497323241238618

**Published:** 2024-05-20

**Authors:** Michael Jauch, Stefano Occhipinti, Analise O’Donovan, Bonnie Clough

**Affiliations:** 1 5723 Griffith University, Brisbane, QLD, Australia; 2 International Research Centre for the Advancement of Health Communication, Department of English and Communication, 26680 Hong Kong Polytechnic University, Hong Kong, China; 3 97562 Griffith Centre for Mental Health, Brisbane, QLD, Australia

**Keywords:** stigma, mental health, health professionals

## Abstract

Mental health professionals stigmatize mental illness, which has significant ramifications for public health and policy. Within this domain, there is a lack of comprehensive research on relative stigma, emotions, and behaviors and an absence of literature that can guide research on these topics. The current study sought to address these limitations. Unstructured interviews were conducted with 22 mental health professionals, and data were analyzed using a grounded theory approach. The current study identified a collection of mental disorders (e.g., borderline personality disorder), stereotypes (e.g., dangerousness), emotion-related responses (e.g., fear), and behaviors (e.g., helping) as being key to the relative stigmatization of mental illness by mental health professionals. The results also suggested that professional context and familiarity with mental illness decrease the stigmatization of mental illness by mental health professionals. These variables and constructs were combined to form a grounded theory of mental health professionals stigmatizing mental illness. The current study has implications for the direction of future research on the stigmatization of mental illness by mental health professionals and interventions that strive to mitigate this type of stigmatization.

## Introduction

There is considerable evidence that at times health professionals stigmatize the people they are meant to help (Chambers et al., 2012; Schulze, 2007; Setchell et al., 2014). For example, some nurses avoid people with HIV/AIDS (Chen et al., 2004), physiotherapists may express a dislike for individuals who are overweight (Setchell et al., 2014), and some physicians negatively stereotype lung cancer patients (Chambers et al., 2012). In the mental health field, mental health professionals can stigmatize people who suffer from mental illness, and this has implications for public health and policy (Chambers et al., 2012; Wahl & Aroesty-Cohen, 2010; World Health Organization [WHO], 2022). The stigmatization of mental illness in general is associated with negative consequences for people with mental illness, and provider-based stigma has a negative impact on health care receivers (Chambers et al., 2012; Overton & Medina, 2008). Further, organizations such as the WHO and the Organisation for Economic Co-operation and Development (OECD) have begun to solicit governments’ involvement to reduce mental illness stigma, including that by mental health professionals (OECD, 2021; WHO, 2022). However, to inform these efforts it is critical that a comprehensive understanding of the stigmatization of mental illness by mental health professionals is developed.

A recent scoping review by Jauch et al. (2023) reported that the literature on mental health professionals stigmatizing mental illness is marked by multiple limitations, including few studies on the relative stigma of mental illness across a variety of mental disorders and little research on the emotional and behavioral dimensions of stigmatization. There is also a lack of literature that can guide research on the mental disorders, stereotypes, emotions, and behaviors that are key to the relative stigmatization of mental illness by mental health professionals. In response, a qualitative study was conducted to examine the relative stigmatization of mental illness by mental health professionals, while accounting for all major components of endorsed stigma (i.e., stereotypes, emotions, and behaviors). The primary aim of this study was to delineate the mental disorders, stereotypes, emotions, and behaviors that are fundamental to the relative stigmatization of mental illness by mental health professionals.

### The Stigmatization of Mental Illness and Its Consequences

Mental illness is stigmatized by the general population in many cultures and contexts (Corrigan, 2005; Hansson et al., 2013). *Stigmatization* can be defined as a collective system of negative reactions that are elicited by human attributes. The components of stigmatization are negative *stereotypes* (e.g., dangerousness), negative *emotions* (e.g., fear), and *discrimination* (e.g., avoidance; Link & Phelan, 2001; Pescosolido & Martin, 2015). When people express agreement with stigmatizing reactions, this is referred to as *endorsed stigma* (Pescosolido & Martin, 2015). From financial difficulties to health problems, the stigmatization of mental illness is related to a variety of negative outcomes for those who suffer from mental illness (Hansson et al., 2013; Schulze, 2007). Stigmatization is one of the main barriers that prevent or delay individuals from accessing mental health care when needed, and this likely increases burden of disease and treatment costs over time (Hansson et al., 2013; Mohr et al., 2010). Recently, the WHO and the OECD made recommendations to improve mental health globally. Of importance was the recommendation that focus be given to the reduction of mental illness stigma (OECD, 2021; WHO, 2022).

Studies further indicate that mental health professionals stigmatize mental illness by endorsing negative stereotypes, emotions, and behaviors (Reavley et al., 2014; Schulze, 2007; Werner & Araten-Bergman, 2017). This type of stigmatization is called *provider-based stigma* (Pescosolido & Martin, 2015). Mental health professionals stigmatizing mental illness, as a specific case of provider-based stigma, is not only inconsistent with expectations about the role of a mental health professional but may also exacerbate the negative outcomes experienced by people with mental illness (Chambers et al., 2012). A recent scoping review identified that there are few studies that extensively investigate the *relative stigmatization* of mental illness by mental health professionals or the degree to which mental disorders are stigmatized compared to other mental disorders (Jauch et al., 2023). Additionally, compared to the number of studies on mental health professionals stereotyping mental illness (i.e., beliefs about mental illness), there is much less consideration given to the emotional and behavioral components of stigmatization (Jauch et al., 2023). Such knowledge will be critical to the development and specification of targets for any evidence-based intervention to reduce the stigmatization of mental illness by mental health professionals. That is, an understanding of relative stigma is needed to identify which interventions should be used for different mental disorder stigmas and which mental disorder stigmas require the most intervention. Further, without knowledge of the emotional and behavioral dimensions of mental health professionals stigmatizing mental illness, interventions will likely overlook important mechanisms of change. Taken together, this knowledge may help inform the mental health services most likely to require additional training or intervention for staff, as well as how interventions may be best delivered.

While a comprehensive understanding of relative stigma, emotions, and behaviors is crucial to reducing the stigmatization of mental illness by mental health professionals, it is unclear as to which mental disorders and aspects of stigmatization should be examined. In the domain of psychopathology, there is a plethora of specific mental disorders and the components of stigmatization present in a number of ways, especially in the case of stereotypes (DSM-5-TR: American Psychiatric Association, 2022; Angermeyer et al., 2011; Corrigan et al., 2003). As such, it is not feasible to conduct research on the stigmatization of mental illness by mental health professionals that captures all mental disorders and instantiations of stigmatization. Therefore, literature is required that can provide direction on which disorders and processes are fundamental. Yet, research on the stigmatization of mental illness by mental health professionals is highly inconsistent in terms of measures of stigmatization (Jauch et al., 2023; Wahl & Aroesty-Cohen, 2010). Adding to this, qualitative studies which could guide research have either placed little emphasis on relative stigma, emotions, and behaviors or were too structured for key disorders and processes to arise from the data naturally (e.g., Burroughs et al., 2006; Clemente et al., 2017; Daibes et al., 2017).

The above qualitative studies also utilized analytical approaches which did not allow for links to be made between variables (e.g., forms of *thematic analysis* that did not make links between constructs), such as the connection in relative stigma between mental disorders and stigmatization. An appropriate solution to this could involve the analytical approach of *grounded theory* (Strauss & Corbin, 1990). Rather than merely summarize observations, grounded theory allows relationships between variables to be found.

### Aims

Accordingly, the current study qualitatively explored the relative stigmatization of mental illness by mental health professionals as it was expressed in the endorsement of stereotypes, emotions, and behaviors. The study’s aim was to identify the mental disorders, stereotypes, emotions, and behaviors essential to the relative stigmatization of mental illness by mental health professionals. Additionally, the current study aimed to generate a theory to outline what these constructs are and how they relate to each other. These aims were achieved through a series of unstructured interviews with mental health professionals and by taking a grounded theory approach to data analysis. It was anticipated that such an approach would go some way to addressing key limitations in the field of mental health professionals stigmatizing mental illness.

## Method

### Interviewer Characteristics and Relationship With Participants

Interviews were conducted by the first author. The interviewer was a male graduate student at Griffith University. Pilot testing was performed by the interviewer, who conducted practice interviews and received feedback from the second author (an experienced researcher and interviewer). Other than email correspondence to organize a time to administer the interview, the interviewer and participants had no relationship prior to the interviews, with the exception that one of the participants was a previous lecturer of the interviewer. Whether previously acquainted or not, all participants knew that the interviewer was operating at Griffith University, and some participants would have been aware that the interviewer was doing the research as part of a PhD dissertation study.

### Study Design

#### Participant Selection

A purposive sampling method was used to select participants, who were deemed eligible if they were a mental health professional aged 18 years or older. Full registration with the Australian Health Practitioner Regulation Agency or an equivalent regulatory agency was required (e.g., Australian Association of Social Workers), unless participants were counselors (for whom registration is not mandated in Australian jurisdictions). The primary goal of sampling was to obtain a selection of representatives spanning a broad range of professions in which mental health services are provided (e.g., psychology, psychiatry, general medicine, psychiatric nursing, occupational therapy, counseling, and social work). Participants were notified of the study by advertisements that were distributed online through the professional networks and personal Facebook pages of research team members, as well as professional Facebook groups and email bulletins. From these sources, participants could access an expression of interest survey that screened for eligibility. Once participants completed this survey, they were informed that they would be contacted via email if they were eligible for further participation.

In total, 22 mental health professionals were interviewed. The sample consisted of seven psychologists, one psychiatrist, one psychiatric registrar, two general practitioners (GPs), three psychiatric nurses, two occupational therapists, three counselors, and three social workers. A fourth psychiatric nurse agreed to participate in the study, but an interview could not be scheduled owing to the individual’s work obligations. This was the only instance of participant dropout. All social workers were registered with the Australian Association of Social Workers, and all other professionals except counselors were registered with the Australian Health Practitioner Regulation Agency. The mean age of the sample was 43.32 years (*SD* = 11.11), and 77.30% of participants identified as female, while the remainder identified as male. Of the sample, 81.80% (*n* = 18) of participants identified as Caucasian, two as Asian, one as Middle Eastern, and one as mixed Caucasian, Asian, and Polynesian ethnicity.

#### Data Collection and Setting

The interview protocol received human research ethics approval from Griffith University. Participants were emailed a consent form, and at the commencement of each interview, the interviewer summarized the study information and gained verbal consent. Before conducting the interviews, an early version of the interview protocol was discussed and role played with expert colleagues who were clinical psychology graduate students and a registered general nurse. Consequently, the interview was revised before again being role played with further graduate students. It was determined that the second version of the interview was sufficient for achieving the aims of the current study and was used as the final interview protocol. All interviews were conducted via the telecommunications application *Zoom*. Data were collected by audio recording the interviews which were later converted into text with the transcription service *Microsoft Azure*. For the interviews, participants were either at their place of residence or in an office at their workplace. The majority of participants were alone for the duration of the interview; however, for one interview the participant’s son was present intermittently.

Interviews began with the interviewer reiterating to the participants that the research team were seeking to better understand how mental health professionals respond to people with mental illness and to do this they would like to ask the participants about their experiences with mental illness. Subsequently, participants were asked “What’s the first thing that comes to mind” and then, once this phase of the interview was over, “What’s it like for you when you are around people with mental illness.” In the final phase of the interview, participants were asked, “Looking back over what we have been talking about, would you have responded any differently with particular mental illnesses.”

By Interview 12, no new major categories were emerging from the data and saturation had been reached. However, at this point in data collection, many responses were indicative of a psychosocial viewpoint, and very few responses were informed by biological or socio-structural perspectives. It was suspected that this may have been the result of not one counselor or GP being interviewed and only one representative being interviewed from psychiatry, psychiatric nursing, and occupational therapy. Thus, to explore stigmatization in contexts where biological and socio-structural frameworks were more likely to manifest, theoretical sampling was utilized to focus data collection on counselors, GPs, psychiatrists, psychiatric nurses, and occupational therapists (Sbaraini et al., 2011). Data collection continued until at least two professionals had been interviewed from each type of mental health profession. Mean interview duration was 32.96 minutes (*SD* = 15.50). Repeat interviews were not carried out with any of the participants, there were no field notes, and transcripts were not returned to participants for their input.

#### Theoretical Framework

The analytical orientation employed in the current study was the grounded theory approach of Strauss and Corbin (1990, 1998). With this framework, researchers formulate novel theory by iteratively classifying qualitative data at varying levels of abstraction. Compared to other forms of grounded theory (e.g., Charmaz, 2014; Glaser & Strauss, 1967), Strauss and Corbin’s grounded theory was chosen because it is an approach that allows for prior knowledge to influence data analysis and provides a systematic procedure for linking constructs.

### Data Analysis and Reporting

The first author read through the first 12 transcripts once to gain familiarity with the data, before independently coding the interviews. Data analysis was verified by agreement with the second author on the coding of five randomly selected transcripts and involved *constant comparative analysis* over three types of coding. First, *open coding* was used to group similar features of the data into preliminary categories or concepts. Once categories were identified with open coding, *axial coding* was utilized to classify the initial categories into higher-order categories that represented types of variables within a theory (i.e., *phenomenon*, *actions/interactions*, *causal conditions*, *contextual conditions*, *intervening conditions*, and *consequences*). Finally, *selective coding* was employed to unify the categories under one core category and to resolve any inconsistencies in the theory. These processes were executed without the use of specialized data management software, and participants were not asked to supply feedback on the results.

As the interview questions were constructed based on existing stigma theory and with the intention of eliciting particular responses from the participants, several categories were expected to arise from the data. It was intended that, in addition to relative stigma, the interview questions would prompt responses revealing positive and stigmatizing reactions to mental illness (i.e., stereotypes, emotions, and behaviors). As such, it was anticipated that these constructs would emerge from the data as broad categories. However, unexpected attributes of the data were still coded, and constructs were only coded if they were present in the data. There were many participant responses, many of which were not relevant to endorsed provider-based stigma. These responses are summarized briefly at the beginning of the Results section but are not included as major categories. Constructs were coded as major categories if they occurred commonly in the data. Quotations are presented in the Results section to illustrate the major categories and subcategories identified with open coding, and quotations are labeled with participant numbers where necessary (e.g., psychologist 1).

## Results

**Figure 1.**
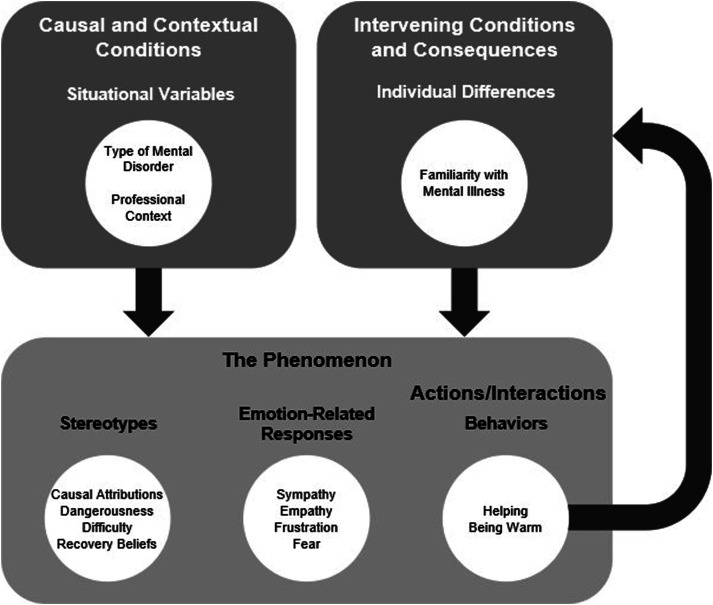
A theory of mental health professionals stigmatizing mental illness.

Figure 1 provides a visual representation of the full grounded theory. The major categories and subcategories derived from the data with open coding are displayed in Table 1. Other than responses that were relevant to endorsed provider-based stigma, participants spoke about various other topics related to mental illness. Some participants responded by talking about systemic barriers to accessing treatment for mental illness and the stigmatization of mental illness by the general population and by other types of professionals. Some participants expressed their beliefs about different treatments for psychopathology and about what constitutes a mental illness.

**Table 1.** Major Categories and Subcategories Identified With Open Coding.

Category/Subcategory	Illustrative Quotes
Stereotypes	
Causal attributions	
Less blame	“I think a lot about socioeconomic factors … I guess I do see a lot of people coming from that social disadvantage and so that’s what I think about, the role and impact of that” (psychiatrist).
	“Whether it’s genetic or whether it’s environmental, I think both contribute” (occupational therapist 2).
More blame	“There’s this resiliency factor that a lot of people don’t seem to have” (social worker 1).
Dangerousness	“Reality is you’re also dealing with a lot of risk … You have to be thinking about their … risk of hurting other people” (psychiatric registrar).
Difficulty	“It’s almost like you’re … dealing with children or teenagers who are just really argumentative and difficult” (psychologist 6).
Positive recovery beliefs	“So look there’s, you know there’s times … you know you actually feel like people are able to make changes in their life” (social worker 3).
Emotion-related responses	
Sympathy	“It can certainly trigger feelings of compassion and sympathy, and also great sadness to be talking with someone and getting an understanding of how difficult it can be for them” (counselor 3).
Empathy	“I think I have a real strong empathy for all them, and struggles that they may be going through” (psychologist 3).
Frustration	“It’s very, it can be really frustrating” (psychiatric nurse 1).

Fear	“It gets to the point where that when there’s people sort of in the street that I come across that … are going through something … that kind of gets a bit of like a fear” (counselor 2).
Not fearful	“It would make my radar go on, but it would not trigger fear” (psychologist 2).
Behavior	
Helping	“Because you want to do the right thing for the person … so that they are able to get some relief from whatever is happening for them” (psychiatric nurse 2).
Being warm	“Just approaching each person with my compassion and empathy. And then just going from there, I guess” (psychiatric registrar).
Relative stigma	“Yeah so, pretty much most of the well I’d say all the patients that are admitted, they’re like acutely psychotically unwell … but I guess then there’s also the I guess personality kind of side of things as well. So you kind of meet them, they’re very unwell and then you kinda gotta wait over time to kind of see what they’re like at baseline, and even at baseline they often still have challenging behaviours … I think when someone’s really acutely psychotically unwell, they’re more unpredictable, whereas versus if it’s like personality like that usually has a pattern of behaviour, so you can kind of predict it in a way” (social worker 2).
	“And it also depends on what mental illness specifically, we talk about something like bipolar ah sorry, borderline personality disorder for example. Hate it. It always makes me cranky yeah. I try to avoid working with it as much as I can. Because I get really frustrated with it. But if we’re talking about something like depression, or anxiety, maybe like depression or if we’re talking trauma type stuff. Then that’s more, there’s a level of calmness” (psychologist 6).
Professional context	“Then in work, sort of, it’s a bit of both in terms of doing what I can and problem solving … and then like I was saying if there’s people that are kind of on the street when I’m not sort of prepared or people that I’m talking to outside of those contexts, I definitely act a lot more scared or like a lot more sort of not wanting to talk about it, not wanting to go into it, sort of ignoring” (counselor 2).
Familiarity with mental illness	“You know if someone came up on the street and you know and I could tell that their, you know they’ve got a mental illness, I wouldn’t be running away on the other side and that sort of side of things, but I think that’s, you know, I think that’s cause I work in the field” (psychiatric nurse 1).

With respect to endorsed provider-based stigma, several participants expressed general positive reactions toward mental illness, and some participants endorsed general stigmatizing reactions toward mental illness. Initially, these reactions were coded as major categories. However, later reading of the transcripts revealed that there were few unambiguously positive or stigmatizing general reactions. More commonly than general reactions, participants conveyed specific reactions to mental illness that consisted of stereotypes, emotion-related responses, and behaviors.

### Stereotypes

Of the specific reactions to mental illness, stereotypes were the most prevalent and often took the form of causal attributions. On the positive end of the causal attribution spectrum, a lot of participants attributed less blame to people for their mental illness. For example, psychologist 1 stated, “I think about how so often mental health is shoehorned into this idea that it’s a health issue, whereas I see it more often as a social issue.” Further, when highlighting how they help clients understand their mental illness, counselor 1 said, “I give them information about the three parts of the brain. So, I focus on the amygdala, the reptilian brain, and the prefrontal cortex.” On the stigmatizing end of the spectrum, a number of participants attributed higher levels of blame to people for their mental illness. For instance, psychologist 5 stated:

My view is that the strategy we adopt is the thing that results in a mental illness diagnosis. So, if my response to my discomfort is to never leave my house, I have agoraphobia. If my response to my discomfort is to be very organized and ordered and make sure everything is as it should be, my diagnosis would be OCD.

Two other common stereotypes were that people with mental illness are dangerous and people with mental illness are difficult. As an example of the dangerousness stereotype, psychologist 6 was asked to elaborate on what they meant by risk and replied, “If we’re talking risk, we’re talking risk to yourself.” Regarding the difficulty stereotype, while talking about counseling over the phone, counselor 3 said, “If there’s an impediment in there due to a mental health condition, then it becomes yeah, challenging and at times exhausting.” The final stereotype that was coded as a major subcategory was recovery beliefs. In particular, almost half the participants expressed positive recovery beliefs through a view that people with mental illness can recover. For example, GP 2 stated, “But you know, you get the people who are then in that recovery phase. They’ve had their acute presentation and they’re, you know, recovering.”

At first, the stereotypes incompetence and unpredictability were also coded as major subcategories. Yet, when seeking illustrative quotes for these stereotypes, instances of misclassification were found. Subsequently, a re-examination of these stereotypes identified that many cases of incompetence were in fact statements irrelevant to stigmatization (i.e., descriptions of the features of mental disorders and an outline of how people in general respond to fear) and unpredictability was more an expression of the dangerousness stereotype.

### Emotion-Related Responses

Among a range of emotion-related responses, two of the most common were sympathy and empathy. For example, psychiatric nurse 1 said, “You talk to them and you’re trying to figure out what’s going on and then you can get that quite, you know, feel a bit sad and feel a bit, you know, upset that they’ve gone through so much,” and the psychiatric registrar commented, “I actually feel like I have a lot of empathy.” On the other hand, there were many instances within the data of stigmatizing emotions. One of the most prevalent was frustration. For example, GP 2, when asked if they had any more feelings to add, stated, “Sometimes it’s just that little bit of frustration.” A number of participants expressed fear toward people with mental illness as well. For instance, occupational therapist 1 said, “So my thoughts and feelings on particular illnesses, mental illnesses, I always find it’s really scary.” However, some participants (including some who expressed fear) stated that they did not fear people with mental illness. For example, psychologist 3 commented “Like I’m not, I’m not scared of it. I’m not intimidated by it. I’m not threatened by it.”

### Behavior

Participants endorsed behaviors toward people with mental illness by either describing their actual behavior or by expressing behavioral intentions. While initially all endorsed behaviors were coded as actual behavior, it became clear later that many of the endorsed behaviors were behavioral intentions. Most of the endorsed behaviors were positive and none of the negative behaviors were coded as major subcategories. Participants frequently endorsed helping people with mental illness and being warm toward them. As an example of helping, social worker 1, when asked if they had any more feelings to add, commented, “Yeah, just those desires you know, the desire to help.” Although being warm was originally a number of more specific codes (e.g., friendly, gentle, and caring), these concepts were ultimately categorized as being warm in general. Occupational therapist 2 demonstrated this behavior by stating “Potentially I think one thing that I do notice is that my behavior will be ah, probably a bit more empathetic, like when I’m around people with mental illness.”

### Relative Stigma and Other Situational Variables

Whether prompted or not, most participants expressed relative stigma at some point in the interview. Further, while participants were either expressing relative stigma or more broadly talking about whether they would have responded differently for particular mental illnesses, the major individual stereotypes, emotion-related responses, and behaviors remained prominent. When explicitly asked if they would have responded any differently to particular mental illnesses, many participants replied in the affirmative. For example, GP 2 replied:

Probably, so the schizophrenics … I also understand that they’re more unpredictable. So, I tend to have some safety things in place with my reception staff. So, we use instant messaging between reception and the doctors in between all the doctors’ rooms, so that we have a code.

Additionally, psychiatric nurse 1 answered:

Yeah, I think I would … I was actually just at a workshop yesterday around eating disorders, borderline personality disorder, and it’s very, it can be really frustrating, and I know some of my behaviors in the past haven’t always been the greatest towards these types of people.

While a large number of participants stated that they would not respond differently for particular mental illnesses, a re-reading of the transcripts uncovered that most had demonstrated relative stigma earlier in the interview.

While either expressing relative stigma or replying to whether they would have responded differently for particular mental illnesses, participants made reference to a range of mental disorders. The main mental disorders were depressive disorders; anxiety and related disorders (e.g., obsessive compulsive disorder and posttraumatic stress disorder); schizophrenia spectrum disorders; bipolar disorder; personality disorders; substance use disorder; and eating disorders (i.e., eating disorder in general and anorexia nervosa). Of the personality disorders, participants frequently referred to personality disorder in general; the most common individual personality disorders were borderline personality disorder and narcissistic personality disorder. Other mental disorders that were noted less frequently included antisocial personality disorder, autism spectrum disorder, dissociative identity disorder, and pedophilic disorder. In regard to the specific pattern of relative stigma, participants were often more positive and less stigmatizing toward depressive disorders and anxiety and related disorders, compared to the other main mental disorders. No other clear pattern of relative stigma was evident within the data.

In addition to relative stigma, there were several other situational variables that emerged from the data as having an impact on stigmatization, although the only one that was coded as a major subcategory was professional context. A number of participants described how they are more positive toward people with mental illness in a professional context in contrast to a personal context, and instances of this were outlined for stereotypes, emotion-related responses, and behaviors. As an example of the effect of professional context, when asked what it is like being around people with mental illness, psychologist 7 said:

Okay, it’s going to depend on where that is. If I’m passing someone in the street … I think I have normal amounts of concern for my own safety … because of the unpredictable nature of someone who’s probably unmanaged and not well stabilized.

When asked how they behave when around people with mental illness, the same psychologist stated, “I’m going to say normal. Yeah, in a professional setting.”

### Individual Differences and Familiarity With Mental Illness

A final type of variable that emerged from the data as having an effect on stigmatization was individual differences. The most common of these variables was familiarity with mental illness. Participants expressed that their professional and personal experiences (including lived experiences) with mental illness were associated with being more positive toward people with mental illness in general and with respect to stereotypes, emotion-related responses, and behaviors. For example, when asked to elaborate on what they meant by their personal experience informing practice, social worker 2 reported how their personal experiences influenced their attitude and empathy toward people with mental illness by saying:

I just try and remember you know what they’re going through, and I guess like, think, reflect on my experience and how I was feeling at the time or how I was reacting at the time and how you know they’re not fully in control of what’s going on … not necessarily consciously, but I guess unconsciously kind of tapping into those experiences and just having a broader understanding as opposed to just kind of judging someone by their behavior or what they’re doing, more kind of considering what’s going on behind that and having more empathy to their situation, yeah.

Another individual differences variable that was initially coded as a major subcategory was type of profession (e.g., a counselor reporting being warm toward people with mental illness as an outcome of being in the field of counseling). However, when gathering illustrative quotes for this variable, it was identified that in most cases the outcome of type of profession was not relevant to stigmatization (e.g., an occupational therapist merely stating that there is more focus on symptoms in occupational therapy).

### A Theory of Mental Health Professionals Stigmatizing Mental Illness

Following the emergence of the major categories and subcategories, these preliminary codes were further classified into higher-order categories. The stereotypes, emotion-related responses, and behaviors were categorized as the phenomenon. This category represents how people respond to the situations in which they find themselves, and in the current study, captured the positive and stigmatizing ways that the mental health professionals reacted to mental illness. Behaviors were also more precisely classified as actions/interactions or the subcategory of the phenomenon that represents what people do either deliberately or habitually to handle the situations in which they find themselves.

Situational variables were coded as causal and contextual conditions because these categories represent the events that influence the phenomenon and the circumstances that people respond to, respectively. By coding the situational variables type of mental disorder (i.e., relative stigma) and professional context as causal and contextual conditions, explanations for the phenomenon were derived (e.g., being in a professional context causes mental health professionals to stigmatize mental illness less). Individual differences were also described by participants as having an effect on stigmatization. However, as such variables are not a collection of events or the situations that people respond to, individual differences were categorized as intervening conditions, which mitigate or alter the impact of causal conditions. Additionally, given that helping and being warm involve contact with people with mental illness, and familiarity with mental illness was reported by participants as resulting from contact, familiarity with mental illness was classified as a consequence of helping and being warm toward people with mental illness, offering an explanation for familiarity with mental illness. At the highest level, these variables were categorized as the situational variables and individual differences that impact how mental health professionals respond to mental illness.

## Discussion

Mental health professionals stigmatize mental illness (Henderson et al., 2014; Schulze, 2007). The current study aimed to delineate the set of mental disorders, stereotypes, emotions, and behaviors that are critical to the relative stigmatization of mental illness by mental health professionals. The current study also aimed to derive a theory that depicted these constructs and the relationships between them.

### Key Findings

A theory was generated on the stigmatization of mental illness by mental health professionals. This theory supplies a novel contribution to the literature through its integration of the three components of stigmatization: relative stigma, other situational variables, and individual differences. At the heart of this theory were the reactions, both positive and stigmatizing, encompassing stereotypes, emotions, and behaviors, that participants expressed toward people with mental illness. These responses are consistent with both existing research on the stigmatization of mental illness by mental health professionals and with the three dimensions of stigmatization outlined by contemporary stigma theory (Carrara et al., 2019; Link & Phelan, 2001; Major & O'Brien, 2005).

The main stereotypes that emerged from the data were causal attributions, varying from less blame to more blame; dangerousness; difficulty; and recovery beliefs, which involved more positive beliefs. The major emotion-related responses that were identified within the data were sympathy, empathy, frustration, and fear. The behavior categories were helping people with mental illness and being warm toward them. While empathy and being warm are frequently overlooked in the literature on mental health professionals (Wahl & Aroesty-Cohen, 2010), many of these responses to mental illness are often examined within research on the general population (Angermeyer et al., 2011; Corrigan et al., 2003; Feldman & Crandall, 2007; Krendl & Freeman, 2019; Pachankis et al., 2018). In particular, several of the above reactions are part of two leading theories of mental illness stigma, *attribution theory* (Weiner, 1995) and the *danger appraisal hypothesis* (Corrigan et al., 2002). Both theories frame discrimination toward people with mental illness as the result of an emotional response that is triggered by a stereotype. The former focuses on causal attributions and sympathy, and the latter emphasizes dangerousness and fear.

One stereotype that was not classified as a major subcategory in the current study was beliefs about the competence of people with mental illness. This stereotype is commonly investigated in research on mental illness stigma (Jauch et al., 2023; Sadler et al., 2015) and is one of the two dimensions in the *stereotype content model* of mental illnesses delineated by Sadler et al. (2012). The absence of this stereotype could have been influenced by where participants tended to offer treatment for mental illness (e.g., community contrasted with non-community settings). However, as the setting that participants worked in was unclear, it was difficult to explain the absence of competence beliefs with the current data, and this is a question that could be addressed in future research. A further striking feature of the results was the lack of participants endorsing avoidance of people with mental illness. Whereas attribution theory emphasizes helping people with mental illness, avoidance is the focus within the danger appraisal hypothesis, and there is a wealth of literature on this form of discrimination (Corrigan et al., 2002; Schulze, 2007). Avoidance may not have been classified as a major subcategory in the current study because mental health professionals often work in contexts where overt avoidance is not possible and more subtle kinds of avoidance are less likely to emerge in qualitative research.

Indeed, context was another crucial aspect of the theory derived in the current study, and numerous participants noted the impact of situational variables on their responses to mental illness. Participants often reported different reactions for individual mental disorders and commonly stated that they were more positive toward people with mental illness in professional contexts than in personal contexts. Although situational variables are frequently excluded from research on mental illness stigma and professional context is completely neglected, these findings are congruent with literature on the relative stigma of mental illness and social psychology’s focus on the *situation* as an important factor in understanding mind and behavior (Deaux & Snyder, 2018; Fiske et al., 2010; Sadler et al., 2012). Granted, there is research on stigma from a social psychological perspective (Major & O'Brien, 2005), but traditionally stigma research has taken a sociological approach (Link & Phelan, 2001; Pescosolido & Martin, 2015). The results of the current study suggest that a social psychological perspective is warranted, and future research could explore the distinction between psychological and sociological approaches.

With the inclusion of relative stigma, the theory generated in the current study is similar to the *behaviors from intergroup affect and stereotypes map* (BIAS map) by Sadler et al. (2015). This BIAS map covers the relative stigmatization of mental illness as it manifests in stereotypes, emotions, and behaviors and, like other BIAS maps, concentrates on the general population (Cuddy et al., 2007; Sadler et al., 2015). The current study provides evidence that the BIAS map framework which has been applied to the general population is also relevant to mental health professionals, despite expectations about their professional role. Further, the proposed theory is the first framework similar to the BIAS map to address the stigmatization of mental illness by mental health professionals.

When expressing relative stigma, participants mentioned a variety of mental disorders. Although many of these are common in research on the relative stigmatization of mental illness by mental health professionals, several are rare in this area of the literature (e.g., borderline personality disorder and eating disorders: Follmer & Jones, 2017; Krendl & Freeman, 2019; Pachankis et al., 2018; Reavley & Jorm, 2011; Schulze, 2007; Wahl & Aroesty-Cohen, 2010). Two mental disorders that were conspicuously missing from the current dataset were antisocial personality disorder and pedophilic disorder. The little research that exists indicates that these mental disorders are generally among the most stigmatized forms of mental illness (Boysen, 2017; Boysen & Logan, 2017; Feldman & Crandall, 2007; Fuss et al., 2018). However, as antisocial personality disorder has a very low prevalence (Lenzenweger et al., 1997), and it is unclear if pedophilic disorder should be labeled a mental illness (Balon, 2013; De Block & Adriaens, 2013), it is possible that these disorders are less salient to mental health professionals.

The last piece of the theory derived in the current study was individual differences. Participants noted several individual differences played a role in how they respond to mental illness; the major individual differences variable was familiarity with mental illness. It was reported by participants that their familiarity with mental illness, both professionally and personally, was related to being more positive toward people with mental illness. The effect of familiarity with mental illness on stigmatization has been investigated extensively and is consistent with literature on the *contact hypothesis* or the notion that intergroup contact reduces prejudice (Angermeyer et al., 2011; Pettigrew & Tropp, 2008).

### Implications

The current study increases our understanding of the mental disorders, stereotypes, emotion-related responses, and behaviors that are fundamental to the relative stigmatization of mental illness by mental health professionals. Additionally, this study highlights professional context and familiarity with mental illness as key to the stigmatization of mental illness by mental health professionals. Although many of these constructs either already have a presence in the research on mental health professionals or are part of existing theory, a number of these constructs have been overlooked within this domain (e.g., empathy, being warm, and borderline personality disorder). Not only do these observations underscore the need for qualitative studies such as the current study, but they also suggest that the methods currently being used in this area to identify key constructs are limited. To this end, the current study provides an empirical basis regarding the mental disorders, stereotypes, emotion-related responses, and behaviors that are important to the stigmatization of mental illness by mental health professionals. As such, the findings of the current study can direct interventions on the mental disorder stigmas that should be prioritized and the variables that should be targeted to bring about change. However, the theoretical framework derived within the current study will likely need to be quantitatively tested and explored, at least partially, before it can be properly utilized by interventions.

### Limitations and Future Directions

The quality of the data within the current study could have been influenced by the social desirability bias associated with stigmatizing mental illness. While this response bias could have been reduced by the confidential nature of the interviews, social desirability bias is inherent to research on the stigmatization of mental illness and may have affected the accuracy of the reports. As another limitation of the current study, participants were not explicitly asked to report whether they deliver mental health services in community or non-community settings. Considering that mental health practice can differ across these contexts, the current study would have benefited from a question that revealed the settings that participants work in.

Future research should rectify these limitations and quantitatively examine the theoretical framework derived within the current study. In particular, the exact relationship between type of mental disorder and each of the three components of stigmatization should be inspected. This should be executed with the mental disorders, stereotypes, emotion-related responses, and behaviors that the current study identified as central to relative stigmatization of mental illness by mental health professionals. Additionally, an extension of this could entail investigating how the stereotypes, emotion-related responses, and behaviors fit together within a causal model. Further, the stability of this model should be investigated with mental health professionals from different levels of experience and training. Such research would help guide interventions at each stage of a mental health professional’s training and ongoing career development. Finally, the current study should be repeated in forensic settings where mental disorders such as antisocial personality disorder and pedophilic disorder have a higher prevalence.

## Conclusion

The current study used interview data to derive a theory of mental health professionals stigmatizing mental illness. Participants responded to the questions about mental illness by endorsing a range of stereotypes, emotion-related responses, and behaviors. Most participants demonstrated the relative stigma of mental illness, and a variety of mental disorders were key to this relative stigma. The current study indicates that for this group of mental health professionals, stigmatization decreases in a professional context, and relative stigmatization is modified by familiarity with mental illness, such that familiarity is associated with more positive reactions. It is hoped that these findings and the proposed theory may further research in this area and inform approaches to reduce stigma among this population. As a likely outcome of this, it is expected that health access and interactions for those experiencing mental illness will be improved.

## Appendix

### Full Interview Schedule

*Introduction*: As outlined in the project information sheets, we are seeking to better understand how mental health professionals respond to people with mental illness. To do this, we would like to ask you about your experience with mental illness.

1. What’s the first thing that comes to mind? … *anything else*?
2. What’s it like for you when you are around people with mental illness? … *anything else*?
3. Looking back over what we have been talking about, would you have responded any differently with particular mental illnesses? … *anything else*?

The following questions can be used as probes should these topics not come up during the interview:

1. What thoughts do you have …? *… anything else*?
2. What feelings do you experience …? *… anything else*?
3. How do you act …? … *anything else*?
